# Hazardous Apoptotic Effects of 2-Bromopropane on Maturation of Mouse Oocytes, Fertilization, and Fetal Development

**DOI:** 10.3390/ijms11114361

**Published:** 2010-11-03

**Authors:** Wen-Hsiung Chan

**Affiliations:** Department of Bioscience Technology and Center for Nanotechnology, Chung Yuan Christian University, Chung Li, Taiwan; E-Mail: whchan@cycu.edu.tw; Tel.: +886-3-265-3515; Fax: +886-3-265-3599

**Keywords:** 2-bromopropane, apoptosis, oocyte maturation, embryonic development

## Abstract

2-Bromopropane (2-BP) is used as an alternative to ozone-depleting cleaning solvents. Previously, we reported that 2-BP has cytotoxic effects on mouse blastocysts and is associated with defects in subsequent development. Here, we further investigate the effects of 2-BP on oocyte maturation and subsequent pre- and post-implantation development, both *in vitro* and *in vivo*. Notably, 2-BP induced a significant reduction in the rates of oocyte maturation, fertilization, and *in vitro* embryonic development. Treatment of oocytes with 2-BP during *in vitro* maturation (IVM) resulted in increased resorption of postimplantation embryos and decreased fetal weights. Experiments with a mouse model disclosed that consumption of drinking water containing 20 μM 2-BP led to decreased oocyte maturation *in vivo* and fertilization *in vitro*, as well as impairment of early embryonic development. Interestingly, pretreatment with a caspase-3-specific inhibitor effectively prevented 2-BP-triggered hazardous effects, suggesting that embryonic impairment by 2-BP occurs via a caspase-dependent apoptotic process. A study using embryonic stem cells as the assay model conclusively demonstrated that 2-BP induces cell death processes through apoptosis and not necrosis, and inhibits early embryo development in mouse embryonic stem cells. These results collectively confirm the hazardous effects of 2-BP on embryos derived from pretreated oocytes.

## 1. Introduction

2-Bromopropane (2-BP), a cleaning agent, is used as an alternative to ozone-depleting solvents. In 1995, 2-BP caused a series of reproductive and hematopoietic disorders in both female and male workers exposed to the material. The solvent was commonly used in an electronics factory located in South Korea [1,2]. Moreover, earlier reports found a high incidence of oligozoospermia in male workers after long-term exposure to 2-BP [1–3]. Several animal studies have further confirmed the potential of 2-BP to injure the reproductive, hematopoietic, central nervous, and immune systems [4–11]. In cytotoxicity experiments, mouse embryos treated with 2-BP displayed micronuclear formation and a decrease in embryo cell number [12]. Moreover, 2-BP was recently identified as a potent DNA damaging agent [5,8]. These results collectively suggest that 2-BP induces various toxicities via DNA damage. A reproductive toxicity investigation further demonstrated that exposure to 2-BP induced testicular or ovarian dysfunction, causing injury to early types of spermatogenic cells or primordial follicles and oocytes of rats [4,6]. In experiments investigating the effects of 2-BP on pre- and postnatal development, exposure of pregnant or lactating female rats to 2-BP resulted in delivery rate decrease, peri- and postnatal death increase, loss of body weight development, and higher incidence of reproductive organ dysfunction [13]. It is true that, to date, no clinical or epidemiological study, or case report, has demonstrated a direct relationship between exposure of pregnant workers to 2-BP and reproductive problems. Specifically, there is no evidence to suggest that the solvent negatively affects embryonic development or infant growth. However, it is very important to explore the health risks associated with exposure of female workers, especially those who are pregnant, to 2-BP. Importantly, the solvent is very volatile and can permeate human skin. The major exposure route is *via* inhalation in the workplace or factory [1,2]. Moreover, a recent study by our group showed that 2-BP induces cellular apoptosis in both the ICM and TE of mouse blastocysts, leading to a decrease in implantation, a reduction in embryonic development, and a loss of embryo viability. These results clearly indicate that 2-BP may be a serious risk factor affecting both the pre- and post-implantation stages of embryonic development. However, both the detailed effects of the solvent, and the precise regulatory mechanisms underlying the potentially adverse effects of 2-BP on oocyte maturation and early embryonic development, require further investigation. In the present study, we determine the effects of 2-BP on mouse oocyte maturation, fertilization, and sequential embryonic development, and next attempt to define the mechanisms involved. Knowledge of the effects of 2-BP on oocyte maturation and fertilization *in vitro* is essential, particularly if pregnant women are to be exposed to the solvent.

Oocyte viability is affected by the microenvironment during growth and maturation. Heat stress, oxygen concentration, and glucose content are key determinants of oocyte viability [14–16]. Several researchers have focused on the influence of environmental biological toxins on oocyte maturation *in vivo* and *in vitro* [17–19]. During normal embryogenesis, apoptosis (a unique morphological pattern of cell death) functions to remove abnormal or redundant cells in preimplantation embryos [20,21]. However, apoptotic processes do not occur prior to the blastocyst stage during normal mouse embryonic development [22], and induction of cell death during oocyte maturation and early embryogenesis (*i.e.*, via exposure to a teratogen) leads to embryonic developmental injury [15,17,23–25]. Previous studies by our group demonstrated that 2-BP promotes cell apoptosis and developmental injury in blastocyst-stage embryos that develop from the zygote for four days [26]. However, the effects of 2-BP on early-stage embryogenesis, such as oocyte maturation, fertilization, and sequential embryo development from zygotes, are currently unclear. Here, we further investigate the effects of 2-BP on oocyte development by incubating with it oocytes for 24 h and comparing sequential development with that of oocytes in 2-BP-free conditions. We aim to determine whether short-term exposure to 2-BP at the oocyte stage has a long-term injurious impact on the pre- and post-implantation stages of embryo development. Our results clearly demonstrate that 2-BP exposure during the oocyte stage not only inhibits oocyte maturation but also promotes injurious effects on *in vitro* fertilization and subsequent embryonic development.

## 2. Results and Discussion

**Figure 1.**
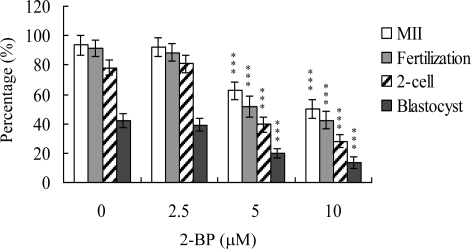
Effects of 2-BP on mouse oocyte maturation and embryo development *in vitro*. Oocytes were collected from 21 day-old mice, cultured for 24 h in IVM medium containing 2-BP (2.5, 5 or 10 μM), fertilized *in vitro*, and transferred to *in vitro* culture (IVC) medium. Oocyte maturation, *in vitro* fertilization, cleavage and blastocyst development were analyzed. Values are presented as means ± SEM of 10 determinations. Data are based on 250–260 samples per group. ***P < 0.001 *versus* the untreated control group.

Recent experiments showed that 2-BP induces apoptosis and developmental injury in mouse blastocysts [26]. However, its effects on mouse oocyte maturation and precise regulatory mechanisms remain to be established. The oocyte nuclear maturation status was measured using 12 independent experimental replicates containing ∼250 oocytes per group. The number of oocytes that reached the metaphase II (MII) stage of maturation after IVM was about 94%. A lower maturation rate was observed in the 5 or 10 μM 2-BP-treated oocyte group, depending on the dose of 2-BP (Figure 1). Male pronucleus formation was assessed for the detection of fertilization. The ability of oocytes to be fertilized by fresh sperm was significantly decreased upon pretreatment with 2-BP, prior to IVM (Figure 1).

We further analyzed *in vitro* embryo development to the two-cell and blastocyst stages. 2-BP pretreatment led to a significant decrease in oocyte cleavage to the two-cell stage, indicative of an injurious effect (Figure 1). In addition, the number of embryos cleaving to form blastocysts in 2-BP-treated groups was significantly lower than that in the untreated control group (Figure 1).

**Figure 2.**
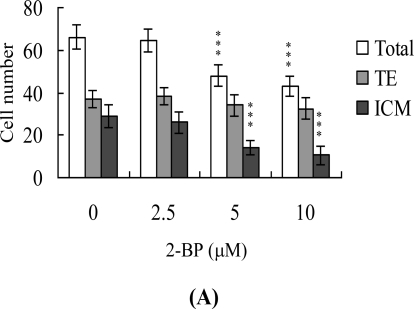

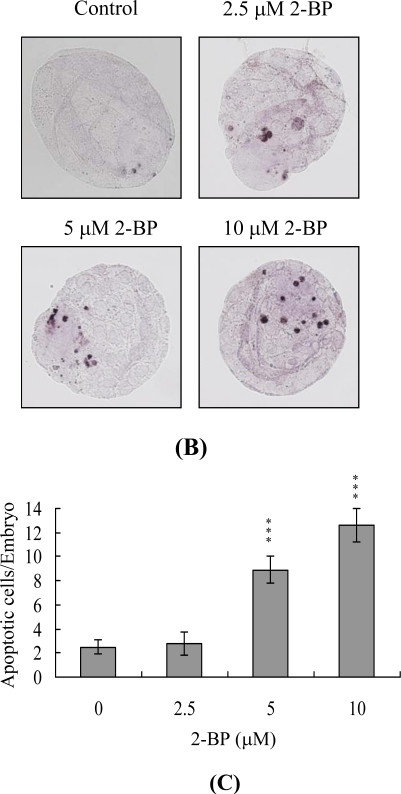
Effects of 2-BP on cell number and apoptosis in embryos during IVM of oocytes. Oocytes were cultured for 24 h in IVM medium containing 2-BP (2.5, 5 or 10 μM), fertilized *in vitro*, and transferred to *in vitro* culture (IVC) medium for *in vitro* development. (A) Cell numbers of total, trophectoderm (TE) lineages and inner cell mass (ICM) were counted in blastocysts. (B) Apoptotic cells were examined at the blastocyst stage using TUNEL staining followed by light microscopy. Positive cells are depicted in black. (C) The mean number of apoptotic (TUNEL-positive) cells per blastocyst was calculated. Values are presented as means ± SEM of eight determinations. Data are based on at least 280 samples in each group. ***P < 0.001 *versus* the untreated control group.

Following 2-BP treatment during IVM of oocytes, total blastocyst cell numbers were counted with a view to establishing its effects on cell proliferation. Differential staining, followed by cell counting, was employed to assess cell proliferation. Significantly lower blastocyst cell numbers were derived from 2-BP-pretreated oocytes, compared to control oocytes (Figure 2A). Additionally, the numbers of ICM cells in blastocysts decreased during IVM after 2-BP pretreatment (Figure 2A). However, 2-BP did not affect the number of trophectoderm (TE) cells present in blastocysts (Figure 2A).

Blastocysts derived from 2-BP-pretreated oocytes was additionally evaluated for apoptosis. TUNEL staining revealed a dose-dependent increase in apoptosis of blastocysts derived from the 2-BP-pretreated oocyte group (Figure 2B). Quantitative analysis further disclosed a 9- to 13-fold increase in apoptotic blastocysts derived from 2-BP-pretreated oocytes, compared to the control group (Figure 2C).

**Figure 3.**
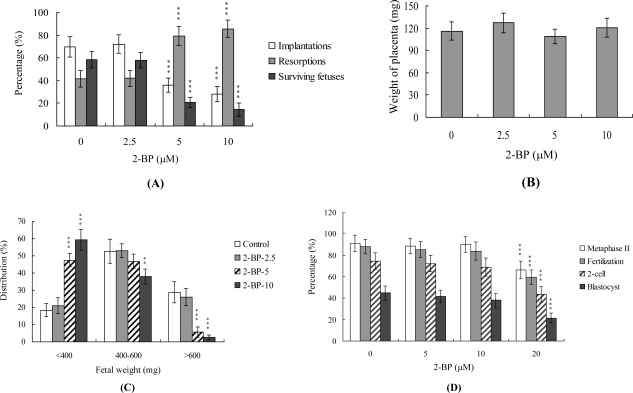
Effects of 2-BP treatment or dietary 2-BP on embryo development during oocyte IVM. Oocytes were cultured for 24 h in IVM medium containing 2-BP (2.5, 5 or 10 μM), fertilized *in vitro*, and transferred to *in vitro* culture medium for development. (A) Implantation, resorption and surviving fetuses were analyzed as described in Materials and Methods. The implantation percentage represents the number of implantations per number of transferred embryos × 100. The percentage of resorption or surviving fetuses represents the number of resorptions or surviving fetuses per number of implantations × 100. (B) Placental weights of 40 recipient mice were measured. (C) Weight distribution of surviving fetuses at day 18 post-coitus. Surviving fetuses were obtained by embryo transfer of control and 2-BP-pretreated groups, as described in Materials and Methods (320 total blastocysts across 40 recipients). (D) Random female mice were fed a standard diet and drinking water supplemented with 2-BP (5–20 μM) for 10 days or left untreated. Oocytes were collected for *in vitro* maturation, *in vitro* fertilization, cleavage, and blastocyst development analyses. Data are based on at least 250 samples in each group. **P < 0.01 and ***P < 0.001 *versus* the 2-BP-free group.

Embryos were transferred to 50 recipients per group (eight per horn). In total, 40 recipients were pregnant in at least one horn at day 18. The implantation ratio of blastocysts derived from the oocyte group treated with 2-BP during IVM was significantly lower than that observed for control blastocysts (Figure 3A).

**Figure 4.**
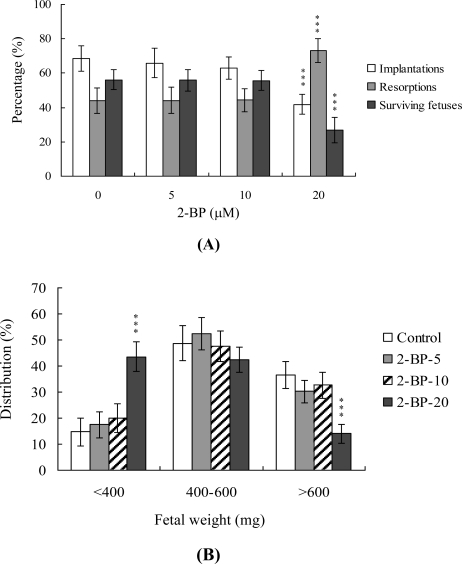
Effects of dietary 2-BP on embryo development in mouse blastocysts. Random female mice were fed a standard diet and drinking water continuously supplemented with 2-BP (5–20 μM) or left untreated for 10 days before embryo transfer to the uterus during the experimental period. (A) Implantation, resorption and surviving fetuses were analyzed, as described in Materials and Methods. The implantation percentage represents the number of implantations per number of transferred embryos × 100. The percentages of resorption or surviving fetuses represent the number of resorptions or surviving fetuses per number of implantations × 100. (B) Weight distribution of surviving fetuses at day 18 post-coitus. Surviving fetuses were obtained via embryo transfer of control and 2-BP intake groups, as described in Materials and Methods (320 total blastocysts across 40 recipients). ***P < 0.001 *versus* the 2-BP-free control group.

To further determine the effects of 2-BP on embryo implantation and post-implantation development, we analyzed 2-BP activity *in vivo* via intake and transfer of blastocyst stage embryos to the uterus horn using the embryo transfer assay in an animal model. Female mice were fed a standard diet and drinking water continuously supplemented with 2-BP (5–20 μM) or left untreated for 10 days before embryo transfer to the uterus during the experimental period. Embryos were transferred to 50 recipients per group (eight per horn). A total of 40 recipients were pregnant at day 18. Notably, the implantation ratio of blastocysts in the 2-BP intake group was significantly lower than that of blastocysts in the control group (Figure 4A). Moreover, the 2-BP intake group displayed a higher overall resorption rate than the control group (Figure 4A). The embryo survival rate of the 2-BP intake group was markedly lower than that of control group (Figure 4A). Interestingly, the placental weights of blastocysts derived from the two groups were not significantly different (data not shown) while fetal weights were lower in the 2-BP intake (20 μM) group (Figure 4B). Based on these results, we conclude that exposure of embryos to 2-BP reduces the potential of embryo implantation and postimplantation development.

**Figure 5.**
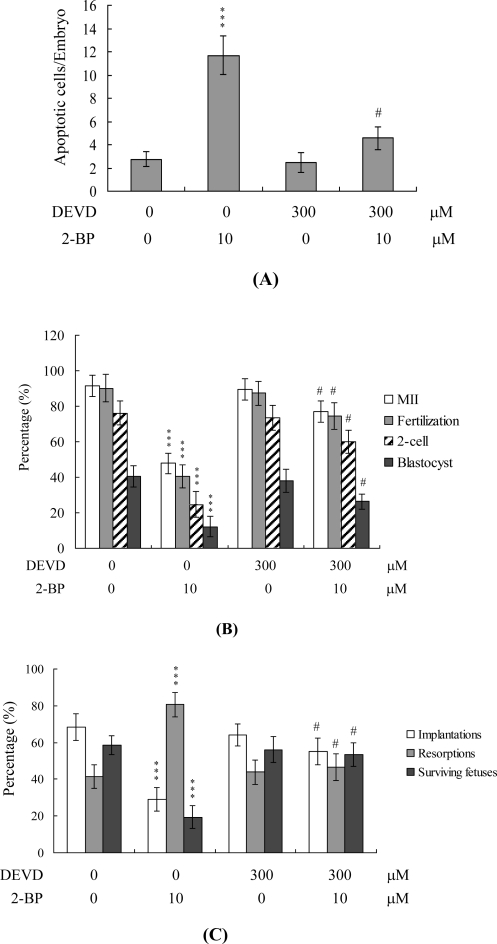
Effects of inhibition of cell apoptosis on embryo development in 2-BP treatment during oocyte IVM. Oocytes were collected, cultured for 24 h in IVM medium alone or containing 100 μM Ac-DEVD-cho and 10 μM 2-BP, fertilized *in vitro*, and transferred to *in vitro* culture (IVC) medium for development. (A) Apoptotic cells were examined at the blastocyst stage by TUNEL staining. (B) Oocyte maturation, *in vitro* fertilization, cleavage and blastocyst development were analyzed. (C) Implantation, resorption, and surviving fetuses were analyzed with the embryo transfer assay, as described for Figure 3. ***P < 0.001 *versus* the untreated control group. #P < 0.001 *versus* 2-BP-treated group.

To further clarify the precise regulatory mechanisms of 2-BP, oocytes were pretreated with 100 μM Ac-DEVD-cho, a caspase-3 specific inhibitor, with the aim of preventing 2-BP-triggered embryo cell apoptosis during IVM of oocytes. Pretreatment with the caspase-3 inhibitor effectively prevented apoptosis of blastocysts derived from the oocyte group pretreated with 10 μM 2-BP (Figure 5A). Notably, the caspase-3 inhibitor blocked 2-BP-triggered hazardous effects on oocyte maturation, fertilization rate, and sequential embryo development during IVM (Figure 5B), and additionally rescued the 2-BP-induced reduction in postimplantation development potential via embryo transfer, including embryo implantation rate, fetal survival rate, and fetal development status (determined on the basis of fetal weight; Figure 5C). These results strongly indicate that the mechanisms underlying 2-BP regulation of oocyte development during IVM involve apoptotic processes.

**Figure 6.**
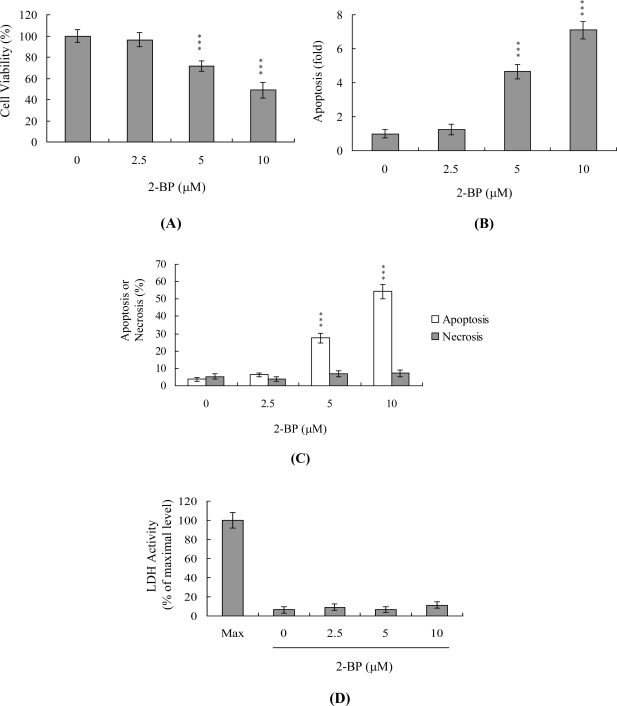
Effects of 2-BP on viability of the mouse embryonic stem cell line, ESC-B5. ESC-B5 cells were treated with the indicated doses of 2-BP for 24 h or left untreated. Cell viability was determined with the MTT assay (A) and apoptosis detected using ELISA (B). (C) Percentages of apoptosis and necrosis were determined by staining cells with propidium iodide and Hoechst 33342. (D) Activity of LDH released in the culture medium of ESC-B5 cells after treatment with various concentrations of 2-BP. Data are expressed as a percentage of the maximal level (Max) of LDH activity determined after total cell lysis. Values are presented as means ± SEM of six determinations. ***P < 0.001 *versus* control (without 2-BP treatment) group.

The numbers of ICM (but not TE) cells in blastocysts decrease during IVM after 2-BP pretreatment (Figure 2). Accordingly, we further investigated the effects of 2-BP on embryonic cell death modes (such as apoptosis and necrosis) and early embryo development and differentiation using the mouse embryonic stem cell line ESC-B5 as the assay model. Mouse ESC-B5 cells were treated with various doses of 2-BP at 37 °C for 24 h, and cell viability and apoptosis measured. As shown in Figure 6A, 2-BP reduced cell viability in a dose-dependent manner. Next, we determined whether 2-BP-induced cell death was attributable to apoptosis. ELISA assays revealed a significant concentration-dependent increase in DNA fragmentation in the presence of 2-BP (Figure 6B). The percentage of apoptotic and necrotic cells was further analyzed by staining with propidium iodide and Hoechst 33342, and necrotic events assessed based on LDH activity in the culture medium. As shown in Figure 6C, the percentage of apoptotic cells increased significantly at 2-BP concentrations higher than 5 μM. Under these conditions, the necrotic cell population remained relatively low (Figure 6C and 6D).

**Figure 7.**
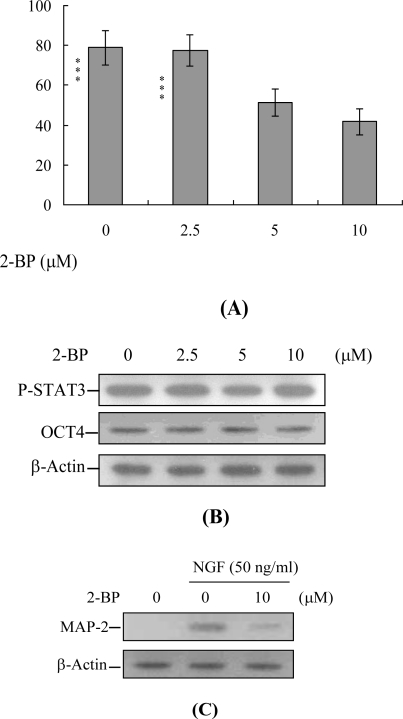
Effects of 2-BP on embryo development, assessed using the embryoid body formation assay. ESC-B5 cells were incubated with the indicated concentrations of 2-BP for 24 h or left untreated. (A) Cells were dissociated with trypsin-EDTA, and cultured in cell medium without LIF to induce differentiation. Embryoid bodies were formed with the hanging method, as described in Materials and Methods. (B) Cell extracts (60 μg) were immunoblotted with anti-p-STAT3 or OCT4 antibodies. (C) ESC-B5 cells were incubated with the indicated concentrations of 2-BP for 24 h or left untreated. Cells were further treated with 50 ng/mL nerve growth factor (NGF) for 14 days. Cell extracts (60 μg) were immunoblotted with anti-microtubule associated protein 2 (MAP-2) antibody. Values are presented as means ± SEM. ***P < 0.001 *versus* the control group.

To further determine the impact of 2-BP on early embryo development in a stem cell assay model, cells were incubated with or without 2-BP and examined for their ability to form embryoid bodies *in vitro*. Embryoid body formation was significantly decreased in cells pretreated with 2-BP (Figure 7A). To ascertain whether the expression levels of OCT 4 and phosphorylated STAT3 (two well-known pluripotent markers) are affected by 2-BP, stem cells were treated with the compound for 24 h or left untreated. Immunoblotting experiments revealed that incubation of mouse ESC-B5 stem cells with 2.5, 5 or 10 μM 2-BP for 24 h had no significant effects on OCT 4 and phosphorylated STAT3 expression, compared to the untreated control group (Figure 7B). Moreover, treatment of embryoid body cells with 50 ng/mL nerve growth factor (NGF) for 14 days induced differentiation into nerve cells, along with expression of microtubule associated protein-2 (MAP-2), a major nerve cell biomarker. Notably, pre-treatment with 10 μM 2-BP effectively inhibited NGF-induced expression of MAP-2 (Figure 7C). These results collectively indicate that 2-BP induces cell death processes specifically via apoptosis and inhibits early embryo development in mouse embryonic stem cells, in keeping with its hazardous effects on embryos derived from 2-BP-pretreated oocytes.

During the complex and precisely orchestrated process of embryonic development, chemical or physical injury can affect normal progression, leading to malformation or miscarriage of the embryo. Thus, it is important to establish the possible teratogenic effects of various chemical agents and environmental toxins. Exposure to 2-BP induces degeneration of germ cells via activation of apoptosis, and spermatogonia are the major target cells [3,27]. Interestingly, 2-BP triggers apoptosis in germ cells through the mitochondria-dependent apoptotic signaling molecules, Bcl-2/Bax, as well as Fas-FasL signaling pathway interactions [27]. In a previous animal study investigating the cytotoxic effects of 2-bromopropane (2-BP) on rat development, rats were exposed to 0–1000 ppm 2-BP via inhalation for 6 h per day, seven days a week for two weeks, prior to mating during the mating period until copulation, and during days 0–19 of gestation. At a concentration of 1000 ppm, inhaled 2-BP significantly suppressed the number of fetuses born, causing fetal lethality during the post-implantation period [28]. Importantly, a recent study by our group showed a marked increase in apoptosis and decreased inner cell mass (ICM) and trophectoderm (TE) cell number in blastocysts treated with 5 or 10 μM 2-BP [26]. Additionally, the implantation success rates of 2-BP-pretreated blastocysts were lower than those of untreated controls. *In vitro* treatment with 5 or 10 μM 2-BP was associated with increased resorption of postimplantation embryos as well as decreased placental and fetal weights [26]. These results strongly indicate that *in vitro* exposure to 2-BP induces apoptosis, suppresses implantation rates after transfer to host mice, and retards early postimplantation development. However, the cytotoxic effects and regulatory mechanisms of 2-BP on oocyte maturation and early-stage embryonic development (such as zygote development to blastocyst) are unclear at present.

Oocyte maturation, fertilization and embryonic development are complex processes during which chemical injury can lead to developmental problems or embryonic malformation. Previous studies report that 2-BP impairs ovarian dysfunction by causing primordial follicle damage and suppressing oocyte maturation in rats. Moreover, 2-BP causes major ovarian dysfunction via induction of cell apoptotic processes to trigger the destruction of primordial follicle and its oocytes [6]. A recent study by our group demonstrated that 2-BP induces apoptosis, impairment of blastocyst development from the morula, and promotion of early-stage death of mouse blastocysts [26]. While 2-BP is clearly a teratogen causing ovarian dysfunction, its effects and precise regulatory mechanisms of action on fertilization and zygote development remain to be ascertained, particularly, short-term exposure of oocytes to 2-BP and long-term early embryo development. It is important to establish the possible teratogenic effects and regulatory mechanisms of 2-BP on oocyte maturation, fertilization, and early-stage embryonic development (such as that from zygote to blastocyst stages). Data from the present study show that 2-BP inhibits mouse oocyte maturation, fertilization, and sequential embryonic development (Figure 1). The 2-BP-pretreated oocyte group displayed a significantly decreased cell number and increased apoptosis (Figure 2A and 2B). Our preliminary HPLC results show that mice exposed to drinking water containing 20 μM 2-BP for four days contain serum 2-BP levels of about 5.63 μM [26]. Clearly, 5–10 μM 2-BP has injurious effects on mouse oocyte maturation, fertilization, and subsequent embryonic development (Figures 1–3). These hazardous effects of 2-BP are evident at treatment doses reflecting physiological concentrations that may be attained through dietary intake. The results collectively indicate that 2-BP treatment at the oocyte stage triggers both oocyte maturation injury and abnormal apoptosis of cells at the blastocyst stage, an important step in embryo implantation.

The TE arises from the trophoblast at the blastocyst stage, and develops into a sphere of epithelial cells surrounding the ICM and blastocoel. These cells contribute to the placenta, and are required for mammalian conceptus development [29]. Reduction in cells of the TE and/or ICM lineage leads to suppressed implantation and lower embryonic viability [30,31]. The ICM and total blastocyst cell numbers are positively correlated with embryonic development during the embryo transfer assay [32]. Application of 2-BP during oocyte maturation had no effect on the TE cell numbers of blastocysts, but led to a dramatic decrease in ICM and total (TE plus ICM) cell numbers (Figure 2). Thus, it appears that 2-BP treatment during IVM causes mortality and/or developmental delay in postimplantation mouse embryos via ICM cell death or decrease in proliferation (Figures 2 and 3). Interestingly, blastocysts derived from 2-BP-treated oocytes displayed decreased implantation, increased embryo resorption, and a lower fetal survival rate (Figure 3A), but comparable placental weight in relation to the control group. TE cells of embryos play important roles in implantation and placental development. Our results indicate that 2-BP does not exert a hazardous effect on TE cells of blastocysts (Figure 2A), and, consequently, has no effect on placental development (Figure 3B). Based on these data, we propose that the decrease in ICM cell number induced by 2-BP during oocyte maturation is the major injurious factor leading to inhibition of embryonic development.

In our experiments, 2-BP-pretreated oocytes displayed significantly decreased fertilization rates and cleavage to the two-cell and blastocyst stages, compared to the untreated control groups (Figure 1), indicative of loss of fertilization and sequent embryonic development. Moreover, in an embryo transfer study, mouse blastocyst stage embryos derived from 2-BP-pretreated oocytes displayed lower implantation and higher resorption rates than control blastocyst-derived untreated oocytes (Figure 3A). Further experiments disclose that the lower embryo implantation rate and fetal weights and higher resorption rate of blastocyst stage embryo transfer to mouse uterus in the 2-BP intake group are significantly different from those of the 2-BP-free control group (Figure 4A and 4B). These results collectively support the theory that 2-BP has potential hazardous effects on early-stage oocyte maturation and fertilization. Importantly, exposure of blastocyst embryos to 2-BP reduces the potential of embryo implantation and postimplantation development.

During embryonic development, cells are often poised between proliferation and apoptosis. We propose that 2-BP can trigger cell death via apoptotic processes (not necrosis) and impair embryo development in a stem cell model (Figures 6 and 7). Incubation of mouse ESC-B5 stem cells with 5 or 10 μM 2-BP for 24 h had no significant effects on the expression of OCT 4 and phosphorylated STAT3, compared to that in the untreated control group (Figure 7B). These results imply that cell apoptosis (Figure 6) and early embryo development injury (Figure 7A), but not pluripotent properties, are affected by 2-BP administered over a short-term period (24 h). In addition, recent experiments by our group showed that 2-BP exerts cytotoxic effects on mouse blastocysts via induction of apoptosis processes [26]. Moreover, 2-BP pretreatment of oocytes led to decreased cell number, apoptosis, and delay in postimplantation development of blastocysts, compared to the control group. These injurious effects were prevented by pretreatment of oocytes with a caspase-3 inhibitor to suppress blastocyst apoptosis (Figure 5). The caspase-3 inhibitor additionally rescued 2-BP-induced reduction in postimplantation development potential following embryo transfer, including embryo implantation rate, fetal survival rate, and fetal development status. Our results indicate that 2-BP triggers improper cell apoptotic processes in early-stage embryos, leading to loss of embryo cell numbers and suppression of post-implantation development, further supporting its role as a teratogen through apoptosis induction properties.

## 3. Experimental Section

### 3.1. Chemicals and Reagents

Dulbecco’s modified Eagle’s medium (DMEM), 2-bromopropane, and pregnant mare serum gonadotropin (PMSG) were obtained from Sigma (St. Louis, MO). Human chorionic gonadotropin (hCG) was purchased from Serono (NV Organon Oss, The Netherlands). TUNEL *in situ* cell death detection kits were acquired from Roche (Mannheim, Germany), and CMRL-1066 medium from Gibco Life Technologies (Grand Island, NY).

### 3.2. COC Collection and in Vitro Maturation (IVM)

ICR mice were acquired from the National Laboratory Animal Center (Taiwan, ROC). This research was approved by the Animal Research Ethics Board of Chung Yuan Christian University (Taiwan, ROC). All animals received humane care, as outlined in the Guidelines for Care and Use of Experimental Animals (Canadian Council on Animal Care, Ottawa, 1984). Mice were maintained on breeder chow (Harlan Teklad chow) with food and water available *ad libitum*. Housing was provided in standard 28 cm × 16 cm × 11 cm (height) polypropylene cages with wire-grid tops, and maintained under a 12 h day/12 h night regimen. Cumulus-oocyte complexes (COCs) were obtained according to a previous protocol [15]. Briefly, COCs were isolated from female hybrid ICR mice (21 days old) injected with 5 IU human chorionic gonadotrophin (hCG) 44 h prior to oocyte collection. COCs were collected in HEPES-buffered α minimum essential medium (MEM) (containing 50 μg/mL Streptomycin sulfate, 75 μg/mL Penicillin G, and 5% fetal bovine serum) by gently puncturing visible antral follicles present on the ovary surface. Germinal vesicle stage oocytes containing an intact vestment of cumulus cells were collected and pooled in at least 10 animals. For oocyte maturation, one drop (∼100 μL) of buffer (αMEM supplemented with 50 μg/mL Streptomycin, 75 μg/mL Penicillin G, 5% FBS and 50 mIU/mL recombinant human FSH) containing 10 COCs was added under oil in 35 mm culture dishes. COC maturation was analyzed following treatment with or without various concentrations of 2-BP (2.5, 5 or 10 μM) for 24 h under an atmosphere of 5% O_2_, 6% CO_2_ and balance of N_2_ at 37 °C.

### 3.3. Maturation Status Assessment

After *in vitro* maturation (IVM), COCs of each group were treated with 50 U/mL ovine hyaluronidase and gently pipetted for the removal of all cumulus cells. Denuded oocytes were collected, and washed with fresh medium, followed by phosphate-buffered saline (PBS). Oocytes were fixed in ethanol: glacial acetic acid (1:3) for 48 h, and stained with 1% aceto-orcein solution. Nuclear structures were visualized using phase-contrast microscopy.

### 3.4. In Vivo Maturation

For obtaining *in vivo* matured oocytes, 21 day-old mice were injected with 5 IU equine chorionic gonadotrophin (eCG) and 5 IU hCG, 61 and 13 h prior to fertilization, respectively. Mature ova were collected from the oviduct into HEPES-buffered α-MEM medium.

### 3.5. Effects of 2-BP Intake on Oocyte Maturation in an Animal Model

The effects of 2-BP on oocytes were analyzed in 21 day-old ICR virgin albino mice. Female mice were randomly divided into four groups of 20 animals each, and administered a standard diet with or without 5–20 μM 2-BP in drinking water for 4 days. COCs were collected by pre-treatment with 5 IU human chorionic gonadotrophin (hCG) for 44 h prior to oocyte collection, and analyzed for oocyte maturation, *in vitro* fertilization, and embryonic development.

### 3.6. In Vitro Fertilization

For *in vitro* fertilization, ova were washed twice in bicarbonate-buffered α-MEM medium (containing 50 mg/mL Streptomycin, 75 mg/mL Penicillin G and 3 mg/mL fatty acid free bovine serum albumin), and fertilized in the same medium with fresh sperm (obtained from a CBAB6F1 male donor). After incubation with sperm for 4.5 h, eggs were washed three times in potassium simplex optimized medium (KSOM) without amino acids in the presence of L-alanyl-L-glutamine (1.0 mM). Next, eggs were placed in 20 mL drops of KSOM under oil, and cultured overnight. During cleavage to the 2-cell stage, embryos were transferred to a fresh drop of KSOM under oil, and cultured for another 72 h. All fertilization steps and embryo culture were additionally carried out under 5% O_2_, 6% CO_2_ and balance of N_2_ at 37 °C.

### 3.7. Fertilization Assessment

For the examination of fertilization, ova were incubated with sperm for 4.5 h, followed by 3 h of culture in fresh medium. Zygotes were assessed for the presence of the male pronucleus with orcein staining, as described previously [15].

### 3.8. Cell Proliferation

Cell proliferation was analyzed by dual differential staining, which facilitated the counting of cell numbers in inner cell mass (ICM) and trophectoderm (TE) [30,33,34]. Blastocysts were incubated with 0.4% pronase in M_2_-BSA medium (M_2_ medium containing 0.1% bovine serum albumin) for the removal of zona pellucida. Denuded blastocysts were exposed to 1 mM trinitrobenzenesulfonic acid (TNBS) in BSA-free M_2_ medium containing 0.1% polyvinylpyrrolidone (PVP) at 4 °C for 30 min, and washed with M_2_ [35]. Blastocysts were further treated with 30 μg/mL anti-dinitrophenol-BSA complex antibody in M_2_-BSA at 37 °C for 30 min, followed by M_2_ supplemented with 10% whole guinea pig serum as a source of complement, along with 20 μg/mL bisbenzimide and 10 μg/mL propidium iodide (PI) at 37 °C for 30 min. The immunolysed blastocysts were gently transferred to slides, and protected from light before observation. Under UV light, ICM cells (which take up bisbenzimide but exclude PI) appeared blue, whereas TE cells (which take up both fluorochromes) appeared orange-red. Since multinucleated cells are not common in preimplantation embryos [36], the number of nuclei represent an accurate measurement of cell number.

### 3.9. TUNEL Assay of Blastocysts

For TUNEL staining, embryos were washed in 2-BP-free medium, fixed, permeabilized, and subjected to labeling using an *in situ* cell death detection kit (Roche Molecular Biochemicals, Mannheim, Germany), according to the manufacturer’s protocol. Photographic images were obtained with a fluorescence microscope under bright-field illumination.

### 3.10. Blastocyst Development Following Embryo Transfer

To determine the ability of expanded blastocysts to implant and develop *in vivo*, embryos generated were transferred to recipient mice. ICR females (6–8 week-old, white skin) were mated with vasectomized males (C57BL/6J; black skin; National Laboratory Animal Center, Taiwan, ROC) to produce pseudopregnant dams as recipients for embryo transfer. To ensure that all fetuses in pseudopregnant mice were derived from embryo transfer (white color) and not fertilization by C57BL/6J (black color), we examined skin color at day 18 post-coitus. To assess the impact of 2-BP on postimplantation growth *in vivo*, COCs were exposed to 0–10 μM 2-BP for 24 h, followed by fertilization and *in vitro* maturation to the blastocyst stage. Subsequently, 8 untreated control embryos were transferred to the left uterine horn, and 8 2-BP-treated embryos to the right uterine horn in day 4 pseudopregnant mice. Forty surrogate mice were analyzed and killed on day 18 post-coitus, and the frequency of implantation calculated as the number of implantation sites per number of embryos transferred. The incidence rates of resorbed and surviving fetuses were calculated as number of fetuses per number of implantations, respectively. The weights of the surviving fetuses and placenta were measured immediately after dissection.

### 3.11. Cell Culture, 2-BP Treatment and Immunoblot

Mouse embryonic stem cells ESC-B5 were cultured in DMEM supplemented with 20% heat-inactivated fetal bovine serum, 100 U/mL penicillin and 100 μg/mL streptomycin. For 2-BP treatment, cells were incubated in media containing various concentrations of 2-BP at 37 °C in a CO_2_ incubator for the indicated time periods. Cells were washed twice with ice-cold PBS and cell lysates were prepared and subjected to immunoblotting as previously described [23,37], using anti-STAT3, Oct4, and MAP-2 antibodies.

### 3.12. Assessment of Necrosis and Apoptosis

Oligonucleosomal DNA fragmentation in apoptotic cells was measured using the Cell Death Detection ELISA^plus^ kit according to the manufacturer’s protocol (Roche Molecular Biochemicals, Mannheim, Germany). Cells (1 × 10^5^) were treated with or without 2-BP at 37 °C for the indicated time periods. Spectrophotometric data were obtained by an ELISA reader at 405 nm. Necrosis was assayed by determining the percentage of cells with plasma membranes permeable to propidium iodide, and apoptosis was assayed by staining with propidium iodide and Hoechest 33342. Cells were incubated with propidium iodide (1 μg/mL) and Hoechest 33342 (2 μg/mL) at room temperature for 10 min. The percentage of apoptotic cells were determined with plasma membrane impermeable to propidium iodide and condensed/fragmented nuclei stained with Hoechst 33342 with fluorescent microscope. In each experiment, 10–12 independent fields (∼600–1000 nuclei in total) were counted per each condition. The activity of lactate dehydrogenase (LDH) present in the culture medium was evaluated as an index of necrosis [38]. Cells were cultured in 96-well microtiter plates (100 μL medium/well) and the absorption values at 490 nm were determined with an ELISA reader according to the manufacturer’s instructions (Promega, Madison, WI). Blanks were carried out adding test substances to medium alone.

### 3.13. Embryoid Body (EB) Formation

Embryoid bodies were formed as previously described [39]. Briefly, ESC-B5 cells were dissociated by trypsin-EDTA (0.25%) and cultured in LIF-free stem cell culture medium to induce differentiation. Cell suspension liquid cultures (<10^4^ cells/mL) were dispensed to 10 cm Petri dishes at 10 mL per dish. Hanging drop cultures were prepared using 10 μL droplets, each containing an appropriate number of ESC-B5 cells for initiation of EB formation. The ESC-B5 cells were allowed to aggregate in the hanging drops for two days, and were then transferred to liquid suspension culture.

### 3.14. Statistical Analysis

Data were analyzed using one-way ANOVA and t-tests, and presented as means ± SEM. Data were considered statistically significant at *P* < 0.05.

## 4. Conclusions

The study results indicate that 2-BP induces developmental injury via induction of cell apoptosis processes in oocyte maturation and early-stage embryos. Our findings clearly suggest that short-term exposure to 2-BP is a risk factor for normal mouse embryonic development, and may inhibit oocyte maturation in infertile subjects.

## References

[1] Kim Y, Jung K, Hwang T, Jung G, Kim H, Park J, Kim J, Park J, Park D, Park S, Choi K, Moon Y (1996). Hematopoietic and reproductive hazards of Korean electronic workers exposed to solvents containing 2-bromopropane. Scand. J. Work Environ. Health.

[2] Park JS, Kim YH, Park DW, Choi KS, Park SH, Moon YH (1997). An outbreak of hematopoietic and reproductive disorders due to solvents containing 2-bromopropane in an electronic factory, South Korea: Epidemiological survey. J. Occup. Health.

[3] Li GX, Kang KS, Lee YS (2001). 2-Bromopropane induced germ cell apoptosis during spermatogenesis in male rat. J. Vet. Med. Sci.

[4] Omura M, Romero Y, Zhao M, Inoue N (1999). Histopathological evidence that spermatogonia are the target cells of 2-bromopropane. Toxicol. Lett.

[5] Zhao LX, Kim EK, Lim HT, Moon YS, Kim NH, Kim TH, Choi H, Chae W, Jeong TC, Lee ES (2002). Synthesis, characterization and in vtro identification of N7-guanine adduct of 2-bromopropane. Arch. Pharm. Res.

[6] Yu X, Kamijima M, Ichihara G, Li W, Kitoh J, Xie Z, Shibata E, Hisanaga N, Takeuchi Y (1999). 2-Bromopropane causes ovarian dysfunction by damaging primordial follicles and their oocytes in female rats. Toxicol. Appl. Pharmacol.

[7] Son HY, Kim YB, Kang BH, Cho SW, Ha CS, Roh JK (1999). Effects of 2-bromopropane on spermatogenesis in the Sprague-Dawley rat. Reprod. Toxicol.

[8] Wu X, Faqi AS, Yang J, Pang BP, Ding X, Jiang X, Chahoud I (2002). 2-Bromopropane induces DNA damage, impairs functional antioxidant cellular defenses, and enhances the lipid peroxidation process in primary cultures of rat Leydig cells. Reprod. Toxicol.

[9] Yu X, Ichihara G, Kitoh J, Xie Z, Shibata E, Kamijima M, Asaeda N, Hisanaga N, Takeuchi Y (1999). Effect of inhalation exposure to 2-bromopropane on the nervous system in rats. Toxicology.

[10] Kim JC, Kim SH, Shin DH, Ahn TH, Kim HC, Kim YB, Jiang CZ, Han J, Chung MK (2004). Effects of prenatal exposure to the environmental pollutant 2-bromopropane on embryo-fetal development in rats. Toxicology.

[11] Ichihara G, Asaeda N, Kumazawa T, Tagawa T, Kamijima M, Yu X, Kondo H, Nakajima T, Kitoh J, Yu IJ, Moon YH, Hisanaga N, Takeuchi Y (1997). Testicular and haematopoietic toxicity of 2-bromopropane, a substitute for ozone layer-depleting chlorofluorocarbons. J. Occup. Health.

[12] Ishikawa H, Tian Y, Yamauchi T (2001). Induction of micronuclei formation in preimplantation mouse embryos after maternal treatment with 2-bromopropane. Reprod. Toxicol.

[13] Kang KS, Li GX, Che JH, Lee YS (2002). Impairment of male rat reproductive function in F1 offspring from dams exposed to 2-bromopropane during gestation and lactation. Reprod. Toxicol.

[14] Sartori R, Sartor-Bergfelt R, Mertens SA, Guenther JN, Parrish JJ, Wiltbank MC (2002). Fertilization and early embryonic development in heifers and lactating cows in summer and lactating and dry cows in winter. J. Dairy Sci.

[15] Banwell KM, Lane M, Russell DL, Kind KL, Thompson JG (2007). Oxygen concentration during mouse oocyte *in vitro* maturation affects embryo and fetal development. Hum. Reprod.

[16] de Castro EPLA, Hansen PJ (2007). Interactions between oxygen tension and glucose concentration that modulate actions of heat shock on bovine oocytes during *in vitro* maturation. Theriogenology.

[17] Chan WH (2009). Impact of genistein on maturation of mouse oocytes, fertilization, and fetal development. Reprod. Toxicol.

[18] Chan WH (2008). Effects of citrinin on maturation of mouse oocytes, fertilization, and fetal development *in vitro* and *in vivo*. Toxicol. Lett.

[19] Malekinejad H, Schoevers EJ, Daemen IJ, Zijlstra C, Colenbrander B, Fink-Gremmels J, Roelen BA (2007). Exposure of oocytes to the Fusarium toxins zearalenone and deoxynivalenol causes aneuploidy and abnormal embryo development in pigs. Biol. Reprod.

[20] Hardy K (1997). Cell death in the mammalian blastocyst. Mol. Hum. Reprod.

[21] Hardy K, Stark J, Winston RM (2003). Maintenance of the inner cell mass in human blastocysts from fragmented embryos. Biol. Reprod.

[22] Byrne AT, Southgate J, Brison DR, Leese HJ (1999). Analysis of apoptosis in the preimplantation bovine embryo using TUNEL. J. Reprod. Fertil.

[23] Chan WH (2006). Ginkgolide B induces apoptosis and developmental injury in mouse embryonic stem cells and blastocysts. Hum. Reprod.

[24] Hsuuw YD, Chang CK, Chan WH, Yu JS (2005). Curcumin prevents methylglyoxal-induced oxidative stress and apoptosis in mouse embryonic stem cells and blastocysts. J. Cell. Physiol.

[25] Shiao NH, Chan WH (2009). Injury effects of ginkgolide B on maturation of mouse oocytes, fertilization, and fetal development *in vitro* and *in vivo*. Toxicol. Lett.

[26] Chan WH (2010). Cytotoxic effects of 2-bromopropane on embryonic development in mouse blastocysts. Int. J. Mol. Sci.

[27] Yu X, Kubota H, Wang R, Saegusa J, Ogawa Y, Ichihara G, Takeuchi Y, Hisanaga N (2001). Involvement of Bcl-2 family genes and Fas signaling system in primary and secondary male germ cell apoptosis induced by 2-bromopropane in rat. Toxicol. Appl. Pharmacol.

[28] Takeuchi T, Okuda H, Arito H, Nagano K, Yamamoto S, Matsushima T (2004). Developmental effects of inhalation exposure to 2-bromopropane in rats. Reprod. Toxicol.

[29] Cross JC, Werb Z, Fisher SJ (1994). Implantation and the placenta: key pieces of the development puzzle. Science.

[30] Pampfer S, de Hertogh R, Vanderheyden I, Michiels B, Vercheval M (1990). Decreased inner cell mass proportion in blastocysts from diabetic rats. Diabetes.

[31] Kelly SM, Robaire B, Hales BF (1992). Paternal cyclophosphamide treatment causes postimplantation loss via inner cell mass-specific cell death. Teratology.

[32] Lane M, Gardner DK (1997). Differential regulation of mouse embryo development and viability by amino acids. J. Reprod. Fertil.

[33] Chan WH (2007). Citrinin induces apoptosis via a mitochondria-dependent pathway and inhibition of survival signals in embryonic stem cells, and causes developmental injury in blastocysts. Biochem. J.

[34] Huang LH, Shiao NH, Hsuuw YD, Chan WH (2007). Protective effects of resveratrol on ethanol-induced apoptosis in embryonic stem cells and disruption of embryonic development in mouse blastocysts. Toxicology.

[35] Hardy K, Handyside AH, Winston RM (1989). The human blastocyst: Cell number, death and allocation during late preimplantation development *in vitro*. Development.

[36] Gardner RL, Davies TJ (1993). Lack of coupling between onset of giant transformation and genome endoreduplication in the mural trophectoderm of the mouse blastocyst. J. Exp. Zool.

[37] Chan WH (2005). Effect of resveratrol on high glucose-induced stress in human leukemia K562 cells. J. Cell. Biochem.

[38] Behl C, Davis JB, Lesley R, Schubert D (1994). Hydrogen peroxide mediates amyloid beta protein toxicity. Cell.

[39] Dang SM, Kyba M, Perlingeiro R, Daley GQ, Zandstra PW (2002). Efficiency of embryoid body formation and hematopoietic development from embryonic stem cells in different culture systems. Biotechnol. Bioeng.

